# Vascular Endothelial Growth Factor (VEGF) Prevents the Downregulation of the Cholinergic Phenotype in Axotomized Motoneurons of the Adult Rat

**DOI:** 10.3389/fnmol.2018.00241

**Published:** 2018-07-12

**Authors:** Lourdes Acosta, Sara Morcuende, Silvia Silva-Hucha, Angel M. Pastor, Rosa R. de la Cruz

**Affiliations:** Departamento de Fisiología, Facultad de Biología, Universidad de Sevilla, Seville, Spain

**Keywords:** VEGF, axotomy, oculomotor system, amyotrophic lateral sclerosis, angiogenesis, laminin, glucose transporter 1 (GLUT-1), acetylcholine

## Abstract

Vascular endothelial growth factor (VEGF) was initially characterized by its activity on the vascular system. However, there is growing evidence indicating that VEGF also acts as a neuroprotective factor, and that its administration to neurons suffering from trauma or disease is able to rescue them from cell death. We questioned whether VEGF could also maintain damaged neurons in a neurotransmissive mode by evaluating the synthesis of their neurotransmitter, and whether its action would be direct or through its well-known angiogenic activity. Adult rat extraocular motoneurons were chosen as the experimental model. Lesion was performed by monocular enucleation and immediately a gelatine sponge soaked in VEGF was implanted intraorbitally. After 7 days, abducens, trochlear, and oculomotor nuclei were examined by immunohistochemistry against choline acetyltransferase (ChAT), the biosynthetic enzyme of the motoneuronal neurotransmitter acetylcholine. Lesioned motoneurons exhibited a noticeable ChAT downregulation which was prevented by VEGF administration. To explore whether this action was mediated via an increase in blood vessels or in their permeability, we performed immunohistochemistry against laminin, glucose transporter-1 and the plasmatic protein albumin. The quantification of the immunolabeling intensity against these three proteins showed no significant differences between VEGF-treated, axotomized and control animals. Therefore, the present data indicate that VEGF is able to sustain the cholinergic phenotype in damaged motoneurons, which is a first step for adequate neuromuscular neurotransmission, and that this action seems to be mediated directly on neurons since no sign of angiogenic activity was evident. These data reinforces the therapeutical potential of VEGF in motoneuronal diseases.

## Introduction

Vascular endothelial growth factor (VEGF) was initially characterized by its actions on the vasculature, inducing vasculogenesis, angiogenesis and increased permeability of capillary vessels (Ferrara and Davis-Smyth, 1997; Yancopoulos et al., 2000). However, evidence has been accumulating during the last years showing that VEGF also exerts direct effects at the neuronal level. For instance, both *in vitro* and *in vivo* experiments have revealed that VEGF acts as a potent neuroprotective molecule when administered to neurons exposed to different types of insults, such as hypoxia, ischemia, excitotoxicity or epileptic seizures (Jin et al., 2000, 2001; Matsuzaki et al., 2001; Lambrechts and Carmeliet, 2006; Nicoletti et al., 2008; Lange et al., 2016). In this regard, a great interest in VEGF has emerged after the recent link established between low levels of this factor and the motoneuronal neurodegenerative disease amyotrophic lateral sclerosis (ALS; Oosthuyse et al., 2001; Sathasivam, 2008), a severe pathology that courses with motoneuronal death and lethality in a few years subsequent to the onset of the disease (Cleveland and Rothstein, 2001; Robberecht and Philips, 2013; Turner et al., 2013).

A crucial study relating VEGF deficit with ALS was obtained by the design of the mutant mice VEGF^δ/δ^, in which the hypoxia response element located in the promoter region of the VEGF gene was deleted, causing a decrease in VEGF levels with time that leads to adult-onset motoneuron degeneration reminiscent of ALS (Oosthuyse et al., 2001). On the other hand, double-transgenic mice resulting from the cross between animals overexpressing VEGF and those presenting a well-accepted model of ALS (superoxide dismutase-1 mutation) display delayed motoneuronal loss, motor improvement, and prolonged survival as compared with the superoxide dismutase-1 mutant mice (Wang et al., 2007). The delivery of VEGF directly or through viral vectors using different routes (intramuscular, intracerebroventricular, intrathecal or into the spinal cord parenchyma) considerably reduces the symptoms of the disease in murine models of ALS (Azzouz et al., 2004; Storkebaum et al., 2005; Dodge et al., 2010; Wang et al., 2016) and also protects motoneurons from excitotoxic-induced cell death (Tovar-y-Romo et al., 2007; Tovar-y-Romo and Tapia, 2010). In this respect, an increase in VEGF and its main receptor (VEGFR-2) has also been described in cerebral motor cortex and hippocampus of postnatal rats following glutamate excitotoxic treatment, which could contribute to neuroprotection mechanisms (Castañeda-Cabral et al., 2017).

Although all these findings indicate that VEGF is a potent neuroprotective factor for vulnerable motoneurons after trauma or disease, nowadays there is lack of information concerning the functional state in which these rescued motoneurons remain. A particular issue of interest is whether VEGF-protected motoneurons continue expressing their neurotransmitter, acetylcholine, an essential step for neuromuscular transmission and motor function. It is well-known that axotomized motoneurons exhibit a downregulation in the expression of the biosynthetic enzyme of their neurotransmitter, choline acetyltransferase (ChAT; Navarro et al., 2007). Low expression of ChAT has also been described in remaining spinal motoneurons of ALS patients in *post-mortem* studies, even at early non-advanced stages (Gillberg et al., 1982; Kato, 1989; Oda, 1999).

Extraocular motoneurons, located in the abducens, trochlear and oculomotor nuclei of the brainstem, constitute a good model to analyze this issue. Indeed, we have previously reported that these motoneurons exhibit a decrease in ChAT content that reaches its maximum at 7 days post-lesion (Morcuende et al., 2005), in agreement with other studies (Navarro et al., 2007). It is important to mention that extraocular motoneurons are more resistant than other cranial or spinal to degeneration in ALS (Reiner et al., 1995; Haenggeli and Kato, 2002). However, they also show signs of degeneration but at later stages of the disease (Takahashi et al., 1993; Sharma et al., 2011; Kimura et al., 2014; Tjust et al., 2017).

In the present work, we aimed at evaluating the ability of exogenously applied VEFG to prevent the loss of the cholinergic phenotype in lesioned extraocular motoneurons of adult rats. Moreover, we have recently demonstrated that extraocular motoneurons express the VEGFR-2, also called Flk1 (Silva-Hucha et al., 2017), the receptor that mainly mediates the neurotrophic effects of VEGF (Oosthuyse et al., 2001; Ogunshola et al., 2002; Brockington et al., 2006; Bogaert et al., 2010; Tovar-y-Romo and Tapia, 2010; Carmeliet and Ruiz de Almodovar, 2013). VEGF administration was carried out peripherally through the proximal stump of the cut nerves innervating the extraocular muscles, given that this factor can travel retrogradely along axons toward the soma (Storkebaum et al., 2005). In the present paper we sought at exploring the angiogenic *versus* the direct retrograde neuroprotective action of VEGF in the lesioned motoneurons.

## Materials and Methods

### Animals and Surgical Procedures

Adult Wistar rats were obtained from an authorized supplier (University of Seville). All experimental procedures were performed in accordance with the guidelines of the European Union (2010/63/EU) and the Spanish legislation (R.D. 53/2013, BOE 34/11370-421) for the use and care of laboratory animals and approved by the local committee for ethics in animal research (Comité Ético de Experimentación de la Universidad de Sevilla). All efforts were made to minimize the number of animals used and their suffering in this study.

A total of *n* = 20 animals were used in the present work: 3 were control non-operated animals, and 17 were lesioned and treated with either vehicle or VEGF. Animals prepared for lesion were operated under general anesthesia (sodium pentobarbital, 35 mg/kg, i.p.). Surgery consisted in the enucleation of the left eye as a method to axotomize extraocular motoneurons, leaving them also deprived of their target muscles. The procedure for enucleation has been described in detail previously (Morcuende et al., 2005, 2011, 2013). Briefly, the left eyeball was extirpated with the help of a ligature made at the back of the eye using a suture thread, which also tied the ophthalmic artery thereby preventing hemorrhage. Intraorbital tissues were also eliminated and finally we cauterized any bleeding originating from the orbital space. The orbit was then filled with an absorbable gelatine sponge pellet (Gelfoam, Pfizer, Belgium) soaked in either vehicle (20 μl of phosphate-buffer saline, pH 7.4, PBS, containing 0.1% bovine serum albumin, BSA), or VEGF (1 μg VEGF prepared in 20 μl of PBS with 0.1% BSA). Sham-operated animals, which received only vehicle, will be referred to as axotomized animals from now onwards. After the implant, eyelids were accurately sutured. The dose used for VEGF administration was within the range applied by other authors (Sun et al., 2003; Manoonkitiwongsa et al., 2004; Storkebaum et al., 2005; Lambrechts and Carmeliet, 2006; Bogaert et al., 2010). We administered the recombinant rat VEGF_164_ aminoacid form that was purchased from R&D Systems (Minneapolis, MN, United States; 564-RV-050). A total of *n* = 5 axotomized and *n* = 6 VEGF-treated animals was used for the study of the cholinergic phenotype in axotomized motoneurons. In addition, we used *n* = 9 animals to test the possibility of an angiogenic side-effect induced by VEGF centrally (3 control, 3 axotomized and 3 treated with VEGF).

A survival time of 1 week was used since, in a previous work, we showed that by this time the downregulation in ChAT was maximal (Morcuende et al., 2005), and also in agreement with previous studies (reviewed by Navarro et al., 2007). Therefore, 1 week after surgery, animals were perfused intracardially under deep anesthesia (sodium pentobarbital, 50 mg/kg, i.p.) with 200 ml of physiological saline followed by 400 ml of 4% paraformaldehyde in PBS. The brainstem was removed, postfixed for 2 h in the same fixative and cryoprotected in a solution containing 30% sucrose in PBS until it sank. The brainstem was then cut coronally at 40 μm thick sections on a cryostat (Leica CM 1850, Wetzlar, Germany). Sections were kept in the fridge at -4°C, immersed in PBS with 0.05% sodium azide, until their processing.

### ChAT Immunostaining

Motoneurons were identified by their immunoreactivity against ChAT. The primary antibody used was a polyclonal goat anti-ChAT IgG (1:500, Merck-Millipore, Darmstadt, Germany; AB-144P). ChAT immunostaining was carried out as follows. First, brainstem sections were incubated for 10 min in a solution containing 10% methanol and 2% H_2_O_2_ in PBS for the inactivation of endogenous peroxidase. After washing in PBS-T (PBS with 0.1% Triton X-100), sections were incubated for 1 h in the blocking solution containing 3% normal horse serum in PBS-T. Tissue was then incubated overnight in the primary antibody solution prepared in PBS-T. After extensive washing, sections were then transferred to the secondary antibody solution in PBS-T for 2 h (biotinylated horse anti-goat IgG, 1:250; Vector Laboratories, Burlingame, CA, United States; BA-9500). Following rinsing, tissue was incubated for 90 min in the avidin-biotin-horseradish peroxidase complex (ABC, Vector Laboratories; PK-6200). Horseradish peroxidase was revealed using 3, 3^′^-diaminobenzidine tetrahydrochloride (DAB) at 0.05% with 0.01% H_2_O_2_ diluted in PBS, followed by nickel intensification of the DAB reaction product by immersing sections in 0.01 mM nickel ammonium sulfate prepared in 0.05 M TRIS buffer, pH = 8. After stopping the reaction with PBS, sections were finally mounted on gelatinized glass slides, dehydrated, cleared in xylene and coverslipped with DPX mounting medium (Panreac, Barcelona, Spain). For controls in this and subsequent immunocytochemistries (see below), we omitted the primary antibody obtaining absence of labeling.

### Evaluation of the Possible Angiogenic Effect of VEGF

In order to check the possibility that VEFG administration could mediate its neuronal effects, at least in part, through its well-known angiogenic activity, we characterized by immunocytochemistry the structure/function of the vascular network in the three extraocular motor nuclei, under the different experimental situations, using the following antibodies (Krum et al., 2002): (i) a marker of the endothelial basement membrane, laminin (polyclonal rabbit anti-laminin, 1:200; Sigma-Aldrich, St. Louis, MO, United States; L 9393); (ii) the glucose transporter 1 (GLUT-1) expressed selectively by endothelial cells of the blood-brain barrier (mouse monoclonal antibody, 1:1000; Merck Millipore, Billerica, MA, United States; MAB S 132); and (iii) the plasmatic protein albumin, as a marker of increased vascular permeability and leakage indicating dysfunction of the blood–brain barrier (sheep polyclonal antibody against rat serum albumin, 1:250; MP Biomedicals, Solon, OH, United States; 55729). Immunoreactivity for these antibodies was carried out in control (*n* = 3), axotomized (*n* = 3) and VEGF-treated (*n* = 3) animals and findings compared between the three groups. To reveal rat serum albumin, a secondary antibody consisting in a biotinylated donkey anti-sheep IgG (Jackson Immuno Research West Grove, PA, United States; 713-065-003) was used at 1:500 following a protocol similar to that carried out for ChAT detection (see above).

Laminin and GLUT-1 were revealed by immunofluorescence in combination with ChAT immunolabeling for the location of the motor nuclei. We performed triple immunofluorescence of ChAT + laminin + GLUT-1 in sequential steps. Using 10% normal donkey serum as the blocking agent, sections were incubated in each primary antibody solution (diluted as indicated above for ChAT, laminin or GLUT-1) and then in its corresponding secondary antibody solution. Secondary solutions contained donkey anti-goat IgG conjugated to Cy2 (1:100; 705-225-003) for ChAT labeling, donkey anti-rabbit IgG conjugated to TRITC for laminin (1:200; 711-025-152), or donkey anti-mouse IgG conjugated to Cy5 (1:200; 715-175-150) to reveal GLUT-1. Rinses were carried out as required with PBS-T, except the last one in which we used PBS. All cytochemical procedures were carried out in free floating sections and at room temperature. Secondary antibodies were obtained from Jackson Immuno Research. After rinsing, sections were mounted on gelatinized glass slides and coverslipped with DAKO fluorescent mounting medium S3023 (Glostrup, Denmark).

Images were captured at the level of the three extraocular motor nuclei with a confocal laser scanning microscope (Zeiss LSM 7 DUO; Oberkochen, Germany) using the 40× objective, in order to analyze and contrast the results obtained between the different experimental situations and motor nuclei. Series of focal planes of 2 μm virtual thickness were captured through the regions of interest. *Z*-stacks were generated after overlapping 6 focal planes of the same histological section by using the Zeiss imaging software ZEN. Analysis was carried out on the *z*-stacks obtained after laminin or GLUT-1 immunostaining. Images were generated in three different fluorescence channels with the following excitation wavelengths: 488 nm for Cy2 (green), 561 nm for TRITC (red), and 633 nm for Cy5 (white).

### Quantification of ChAT Immunostaining in Motoneurons

To count the number of motoneurons, we used the 40-μm transverse brainstem sections labeled with the anti-ChAT antibody. We estimated by stereological methods the number of ChAT-positive cells in the abducens, trochlear and oculomotor nuclei using the optical fractionator method (Golub et al., 2015) by means of the newCAST stereological software (Visiopharm, Hoershold, Denmark) adapted to an Olympus BX61 microscope (Hicksville, NY, United States). Countings were performed at both the left and right sides of each nucleus in axotomized (*n* = 5) as well as VEGF-treated (*n* = 6) animals. The principle of Cavalieri was used to estimate the volume of the extraocular nuclei. The number of neurons in the analyzed region was estimated by multiplying the number of neurons counted within the sample regions by the reciprocals of the volume sampling fraction (De Pablos et al., 2014). Data were normalized per nucleus and animal and expressed as percentage values relative to the control side.

Since abducens motoneurons project ipsilaterally and superior oblique motoneurons contralaterally to their corresponding extraocular muscles, then the affected side of the abducens and trochlear nuclei was the left and the right side, respectively, after left eye enucleation. The oculomotor nucleus contains four motoneuronal subdivisions (three project ipsilaterally and one contralaterally) with a similar number of motoneurons per subdivision (Glicksman, 1980; Miyazaki, 1985; Evinger, 1988). Therefore, for ChAT countings, we applied a correction as previously described (Morcuende et al., 2005, 2011, 2013) to consider the left oculomotor nucleus as the affected one. Briefly, we solved the following equation: 3*x* + *y* = *n_1_* and 3*y* + *x* = *n_2_*, where *n_1_* and *n_2_* are the number of oculomotor motoneurons ipsilateral and contralateral, respectively, to the lesion side (left). Therefore, we calculated *x* and *y*, where *x* is the number of motoneurons per lesioned subnucleus (three subdivisions ipsilateral and one contralateral) and *y* is the number of motoneurons per control subnucleus.

We also quantified the intensity of ChAT immunostaining. We measured the mean gray value (optical density) of the motoneurons in the three nuclei, at both sides, and in the two experimental groups (i.e., axotomized and VEGF-treated) by means of the Image J program (NIH, Bethesda, MD, United States). The outline of each ChAT-positive motoneuron within the limits of each nucleus was traced (the cell nucleus excluded), and the program gave the mean gray value per cell (intensity of cell immunostaining). To normalize data among different sections, cell values were divided by the background level obtained in the same section. Finally, data were normalized per nucleus and animal, and expressed as percentage values relative to the control side.

### Quantification of Blood Vessel Immunostaining and Vascular Permeability

The analysis of laminin and GLUT-1 optical density in the abducens, trochlear and oculomotor nuclei, was carried out using the *z*-stacks described above, generated by the confocal microscope software. The boundaries of the three motor nuclei were determined by the ChAT immunolabeling. Images were analyzed with the help of the Image J program. For measurements, a grid was elaborated consisting in squares of 10 μm × 10 μm spaced every 10 μm. Within each square we measured the mean gray value, excluding those containing a ChAT-immunopositive cell. Only those squares showing an optical density value higher than 0.1, after background subtraction, were computed for the analysis. Then we calculated the percentage of 10 μm × 10 μm squares whose optical density was higher than 0.1 in relation to the total number of squares counted per section. Data were measured in control (*n* = 3), axotomized (*n* = 3), and VEGF-treated (*n* = 3) animals on the affected side of each nucleus or, in the case of control animals, on the left side. For axotomy and VEGF treatment, data were normalized per nucleus and animal, and expressed as percentage relative to the control animals.

Since GLUT-1 labels mature endothelial cells (Krum et al., 2002), we also quantified the area in those blood vessels that were laminin-immunopositive but GLUT-1-immunonegative, as a likely index of newly-formed vessels, by outlining the region of interest with the help of the program Image J. This analysis was performed in the triple immunofluorescence sections within the limits of each motor nucleus, identified by the ChAT immunostaining. Data were measured in control (*n* = 3), axotomized (*n* = 3), and VEGF-treated (*n* = 3) animals on the affected side of each nucleus or, in the case of control animals, on the left side. Finally, data were normalized per nucleus and animal, and expressed as percentage values relative to the control animals.

In the case of albumin immunostaining, we also measured the optical density (with the help of the Image J program) in the affected side of each motor nucleus for the axotomized (*n* = 3) and VEGF-treated animals (*n* = 3), as well as in the left side for control intact animals (*n* = 3). Optical density was compared between control, axotomized or VEGF-treated nuclei.

### Statistics

Data were represented as the mean and standard error of the mean (SEM). The number of ChAT-positive cells as well as the intensity of the different immunostainings were compared between the different extraocular motor nuclei (factor 1) and experimental situations (factor 2). To detect differences between groups, the two-way ANOVA test was used at an overall level of significance of 0.05 followed by the *post hoc* Holm–Sidak method for all pairwise multiple comparisons. When required, we also used the paired *t*-test for comparisons between the two sides of the same nucleus. Statistics was performed by using the program SigmaPlot 11 (Systat Software, Inc., Chicago, IL, United States).

## Results

### Axotomized Extraocular Motoneurons Exhibit a Downregulation in ChAT Expression

**FIGURE 1:**
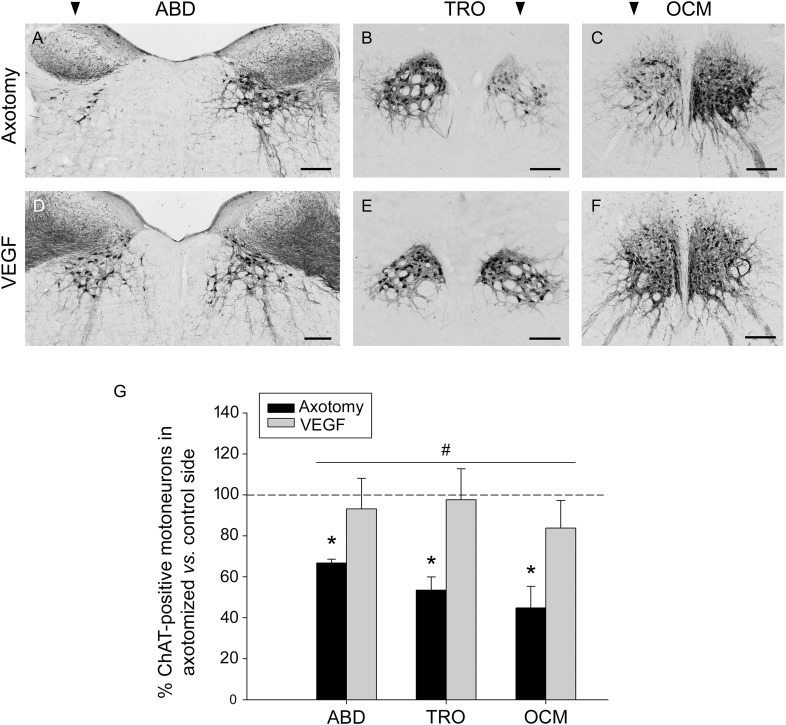
Vascular endothelial growth factor (VEGF) prevents the loss of the cholinergic phenotype in lesioned extraocular motoneurons. Images of coronal sections through the abducens (ABD), trochlear (TRO), and oculomotor (OCM) nuclei of axotomized animals treated with either vehicle **(A–C)**, or VEGF **(D–F)**. Sections were immunostained against ChAT, the biosynthetic enzyme of the motoneuronal neurotransmitter acetylcholine. The affected side of each nucleus is indicated by an arrowhead at the top of **(A–C)**. Note the marked reduction in the number of ChAT-immunolabeled motoneurons in the affected side of axotomized animals as compared with their respective control side **(A–C)**. In contrast, VEGF administration maintained, in the axotomized side, a number of ChAT-immunostained motoneurons similar to the control side (**D–F**; compare control with treated side). Scale bars = 200 μm. **(G)** Bar chart illustrating the number of ChAT-positive motoneurons in axotomized (black) and VEGF-treated (gray) animals for the three extraoculomotor nuclei (ABD, abducens; TRO, trochlear; OCM, oculomotor) expressed as the percentage of immunostained motoneurons in the affected side relative to the control side. The horizontal dashed line indicating 100% refers to the control side of each nucleus, used to normalize data (^∗^*p* < 0.05, significant differences with respect to the control side, paired *t*-test). Note that the number of ChAT-immunoreactive motoneurons was significantly higher in VEGF-treated animals as compared to axotomized animals (treated with vehicle) for ABD, TRO, and OCM. In turn, no significant differences were found between these nuclei irrespective of the treatment. Two-way ANOVA (factor 1, nucleus; factor 2, treatment) followed by Holm–Sidak method for all pairwise multiple comparisons. ^#^*p* < 0.05, significant differences between axotomy and VEGF treatment. Data represent mean and SEM; *n* = 5 axotomized and *n* = 6 VEGF-treated animals for each nuclei.

The axonal section of extraocular motoneurons performed at the orbital level led to a noticeable decrease in the immunostaining against ChAT in the corresponding brainstem motor nuclei (abducens, trochlear, and oculomotor). The examination of the immunostained sections clearly showed that the number of ChAT-positive motoneurons in the lesioned side of these nuclei (arrowhead in **Figures 1A–C**) was markedly lower than that observed in their respective control side. Thus, the number of stained cells fell to 66.74 ± 1.89%, 53.48 ± 6.45%, and 44.83 ± 10.56% (with respect to the control side) in the abducens, trochlear, and oculomotor nuclei, respectively, 1 week after lesion (**Figure 1G**). The statistical comparison of the number of ChAT-immunoreactive motoneurons between the control and the axotomized side revealed significant differences in the abducens (*p* < 0.001, paired *t*-test), trochlear (*p* = 0.021, paired *t*-test) and oculomotor (*p* = 0.01, paired *t*-test) nuclei (*n* = 5 animals), in all cases with a significantly lower number of ChAT-immunoreactive motoneurons in the axotomized side (**Figure 1G**, asterisks).

**FIGURE 2:**
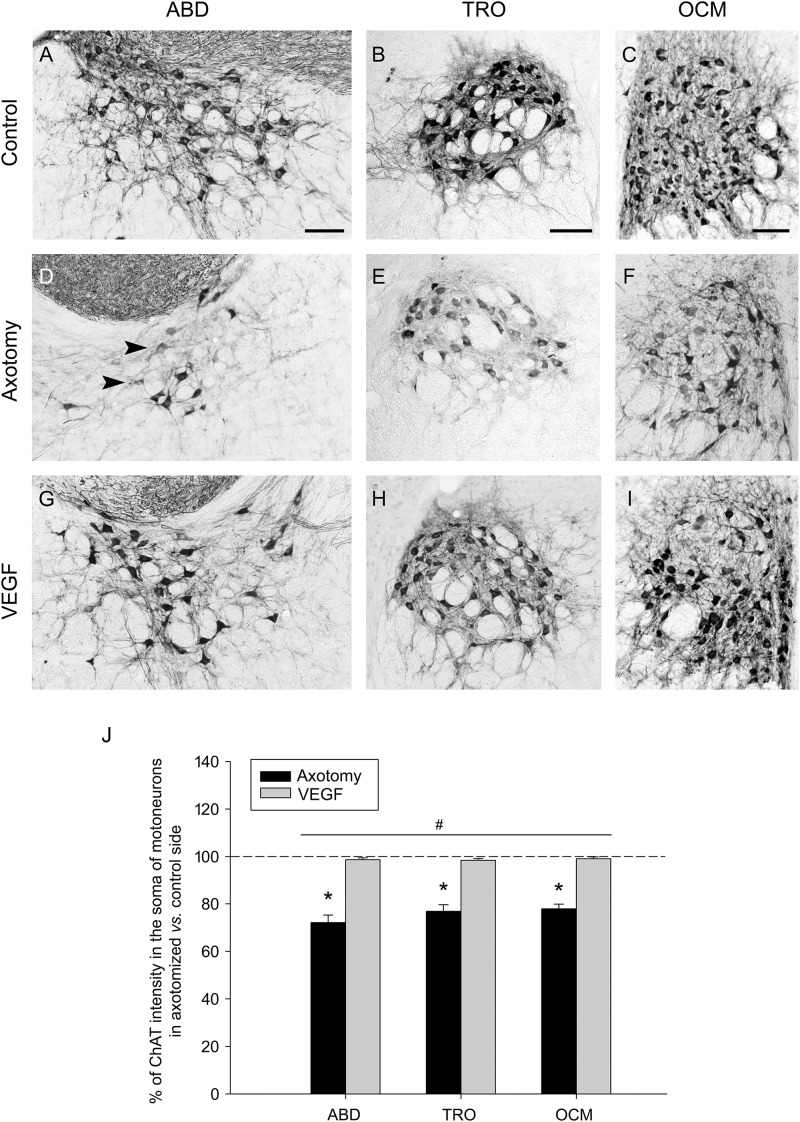
Vascular endothelial growth factor maintains a normal intensity of ChAT immunostaining in axotomized motoneurons. **(A–C)** ChAT immunolabeling in the control side of the abducens (**A**, ABD; right side), trochlear (**B**, TRO; left side) and oculomotor (**C**, OCM; right side) nuclei. **(D–F)** Same as (**A–C)** but for the lesioned side (**D**, left ABD; **E**, right TRO; **F**, left OCM) of an axotomized animal. **(G–I)** Same as **(D–F)** but for a VEGF-treated animal. Note the presence of faintly-labeled cells in the axotomized size **(D–F)** of lesioned animals (arrowheads). In contrast, ChAT immunoreactive-cells in VEGF-treated animals **(G–I)** showed a staining intensity similar to control **(A–C)**. Scale bars = 100 μm. **(J)** Bar chart illustrating the percentage of ChAT immunostaining intensity in motoneuron cell bodies of axotomized (black columns) and VEGF-treated (gray columns) animals for the three extraoculomotor nuclei (ABD, TRO and OCM), expressed as the percentage of ChAT labeling intensity in the affected side with respect to the control side. The horizontal dashed line at 100% level refers to the control side of each nucleus, used for normalization of data (^∗^*p* < 0.01, significant differences with respect to the control side, paired *t*-test). Note that the intensity of ChAT-immunoreactive motoneurons was significantly higher in VEGF-treated animals as compared to axotomized animals (treated with vehicle) for ABD, TRO, and OCM. In turn, no significant differences were found between ABD, TRO, and OCM irrespective of the treatment. Two-way ANOVA (factor 1, nucleus; factor 2, treatment) followed by Holm–Sidak method for all pairwise multiple comparisons. ^#^*p* < 0.05, significant differences between axotomy and VEGF treatment. Data represent mean and SEM. *n* = 5 axotomized and *n* = 6 VEGF-treated animals for each nuclei.

We also observed that ChAT immunolabeling in the axotomized side showed, in comparison with the control side, not only a fewer number of labeled cells but also the presence of weakly-stained motoneurons, so that the affected side was characterized by a faint staining (**Figures 2D–F**) that markedly contrasted with the strong labeling present in the control side (**Figures 2A–C**). For this reason, we also quantified the intensity of immunolabeling by measuring the optical density within the cytoplasm of ChAT-positive motoneurons and expressed data as percentage relative to the control side. In axotomized animals, the optical density of ChAT immunolabeling decreased to 72.21 ± 1.94%, 76.90 ± 1.94%, and 77.89 ± 1.43% (as compared to the control side; *n* = 5) in the abducens, trochlear and oculomotor nuclei, respectively. The statistical comparison of the intensity of ChAT immunostaining between the control and the axotomized side revealed significant differences in the abducens (*p* = 0.001, paired *t*-test), trochlear (*p* < 0.001, paired *t*-test), and oculomotor (*p* < 0.001, paired *t*-test) nuclei (*n* = 5 animals), with a significantly lower level of ChAT intensity in the axotomized side of the three nuclei (**Figure 2J**, asterisks).

Therefore, axotomy produced a significant decrease in both the number of ChAT-immunoreactive motoneurons and the intensity of ChAT immunostaining.

### VEGF Administration Prevents the Downregulation Exhibited by Axotomized Motoneurons

An interesting finding of the present work was that treatment with VEGF at the time of axotomy prevented the reduction in ChAT immunostaining caused by lesion, so that the affected side (axotomized plus VEGF-treated) appeared with a similar number of ChAT-immunolabeled motoneurons as compared with the control side (**Figures 1D–F**). The statistical analysis confirmed our observations revealing absence of significant differences in the abducens, trochlear, and oculomotor nuclei between the treated *versus* the control side for those animals that received the VEGF intraorbital implant (paired *t*-tests; **Figure 1G**, *n* = 6). Furthermore, we also compared the percentages of ChAT-positive motoneurons in the affected side between axotomized and VEGF-treated animals after normalization to their respective control side (two-way ANOVA test followed by Holm–Sidak method for *post hoc* comparisons; **Figure 1G**). VEGF treatment showed a significantly higher percentage of ChAT-immunoreactive motoneurons (93.16 ± 14.94% for abducens, 97.61 ± 15.14% for trochlear, and 83.77 ± 13.49% for oculomotor) than in the axotomy condition (see above the axotomy percentages; *p* < 0.05; **Figure 1G**, hashtag, compare gray columns with black columns). In turn, there were no significant differences between nuclei irrespective of treatment.

Vascular endothelial growth factor also protected motoneurons against the decrease in the level of ChAT immunostaining induced by lesion. Thus, motoneurons of VEGF-treated animals (**Figures 2G–I**) appeared markedly-stained in a similar way to those of the control side (**Figures 2A–C**). Indeed, we obtained statistical similarity regarding ChAT intensity in the abducens, trochlear, and oculomotor nuclei between the VEGF-treated *versus* the control side (**Figure 2J**, paired *t*-tests; *n* = 6). Furthermore, we compared the percentages of ChAT immunostaining intensity in motoneurons of the affected side between axotomized and VEGF-treated animals after normalization to their respective control side (two-way ANOVA test followed by Holm–Sidak method for *post hoc* comparisons; **Figure 2J**). VEGF treatment produced a significantly higher percentage of ChAT optical density (98.58 ± 1.43% for abducens, 98.33 ± 1.37% for trochlear, and 99.01 ± 1.12% for oculomotor) than in the axotomy condition (see above the axotomy percentages; *p* < 0.05; **Figure 2J**, hashtag, compare gray columns with black columns). The test also revealed absence of significant differences between abducens, trochlear, and oculomotor nuclei irrespective of treatment.

Therefore, these data confirmed that VEGF delivery to injured motoneurons protected them against the downregulation in ChAT expression, in regard to both the number of ChAT-positive motoneurons and the intensity of ChAT immunostaining.

### The Neuronal Effects Mediated by VEGF Were Not Associated to an Angiogenic Response

Due to the well-known effects of VEGF on the vasculature, including angiogenesis and increased vascular permeability, we aimed at elucidating whether the effects found at the neuronal level after VEGF administration could be explained by its action on the vascular system, that is, providing more nutrients and oxygen to the affected motoneurons and in this way promoting neuroprotection or, alternatively, whether VEGF might act directly on neurons. For this purpose, and in accordance with previous studies (Oosthuyse et al., 2001; Krum et al., 2002; Azzouz et al., 2004), we performed an immunohistochemical study against the following antigens: (i) albumin, a plasmatic protein used as an index of vascular leakage induced by increased permeability; (ii) laminin, as a marker of the basement membrane of blood vessels; and (iii) GLUT-1, for the specific labeling of endothelial cells. In these experiments we used three different groups of experimental animals: (i) intact, untreated, controls (*n* = 3); (ii) axotomized and treated with vehicle (*n* = 3; axotomy condition); and (iii) axotomized but treated with VEGF (*n* = 3; VEGF condition).

**FIGURE 3:**
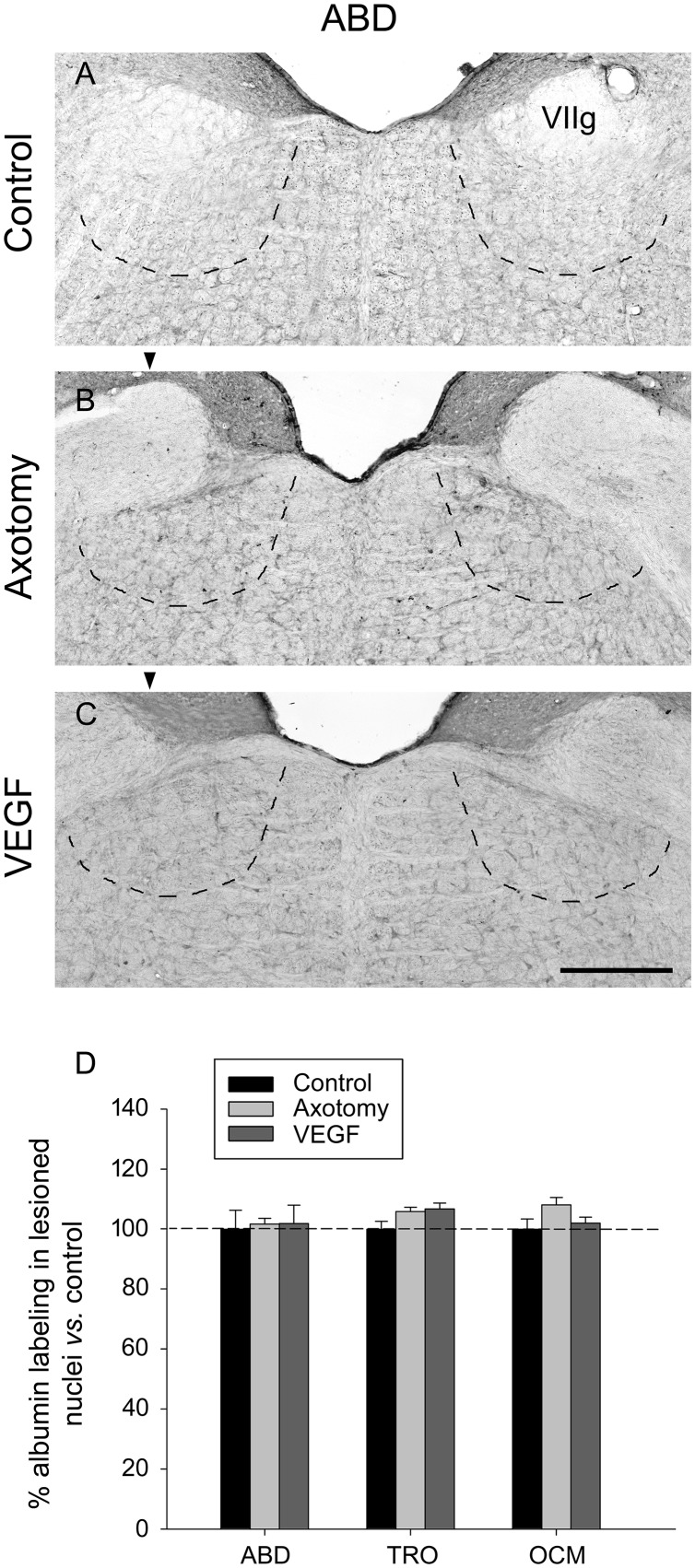
Vascular endothelial growth factor administration does not produce vascular leakage as revealed by rat serum albumin immunostaining. Images of coronal sections through the abducens nucleus showing virtually absence of albumin immunoreactivity in the three experimental situations: **(A)**, control intact animal; **(B)**, axotomized animal treated with vehicle; and **(C)**, axotomized animal treated with VEGF. Arrowheads in **(B,C)** point to the affected (axotomized) side. The dashed lines in **(A–C)** label the boundaries of the abducens nucleus. VIIg, genu of the facial nerve. Scale bar = 200 μm. **(D)** Bar chart illustrating the measurements of albumin immunolabeling in the different experimental situations (control, axotomized and VEGF-treated animals) and for the three extraocular motor nuclei (ABD, abducens; TRO, trochlear; and OCM, oculomotor nuclei). Data represent mean ± SEM and are expressed relative to the control animals (100%, horizontal dashed line). Comparisons were carried out by means of the two-way ANOVA test at a level of significance of *p* < 0.05. Note absence of significant differences between nuclei and treatments. *n* = 3 animals in each situation and nucleus.

The immunostaining of albumin was carried out at the light microscopy level in the different extraocular motor nuclei and situations using DAB as the chromogen. The visualization of sections revealed in all cases a very faint and diffuse staining that did not label any particular element within the tissue (**Figures 3A–C**, abducens nucleus illustrated). The measurements of optical density in each nucleus after albumin immunostaining revealed that there were no significant differences between either the different nuclei or situations (**Figure 3D**, two-way ANOVA test followed by Holm–Sidak method for *post hoc* comparisons). Therefore, our data of albumin immunolabeling suggested absence of significant increase in vascular permeability due to VEGF treatment.

**FIGURE 4:**
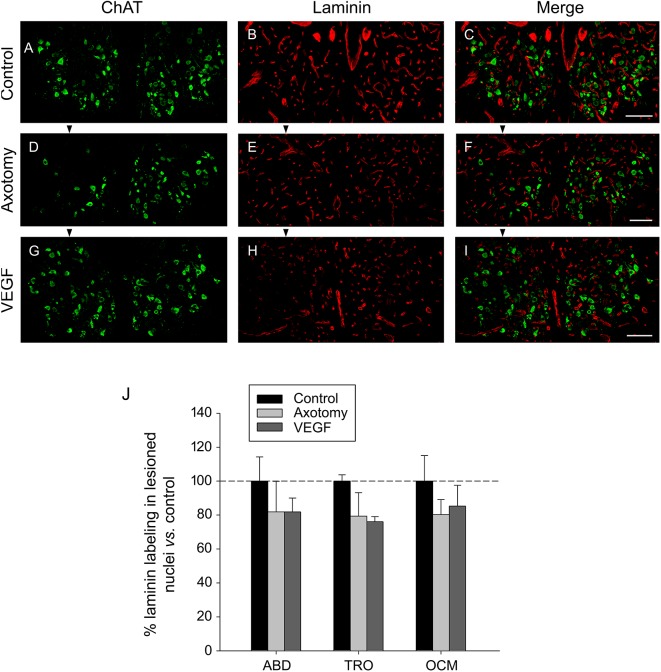
Laminin immunostaining reveals absence of VEGF-induced angiogenesis. Confocal microscopy images of coronal sections through the oculomotor nucleus against ChAT and laminin showing absence of angiogenesis after VEGF administration. **(A–C)** Control intact animal. **(D–F)** Axotomized animal treated with vehicle. **(G–I)** Axotomized animal treated with VEGF. Arrowheads point to the affected (axotomized) side in **(D–I)**. Motoneurons were identified as ChAT-immunoreactive (**A,D,G**, in green). Laminin was used as a marker of blood vessels (**B,E,H**, in red). The merged images of ChAT and laminin are illustrated in **(C,F,I)**. Note a similar density of laminin-labeled blood vessels surrounding motoneurons in the three experimental situations. Scale bars = 100 μm. **(J)** Bar chart illustrating the measurements of laminin immunoreactivity in the different experimental situations (control, axotomized, and VEGF-treated animals) and for the three extraocular motor nuclei (ABD, abducens; TRO, trochlear; and OCM, oculomotor nuclei). Data represent mean ± SEM and are expressed relative to the control animals (100%, horizontal dashed line). Comparisons were carried out by means of the two-way ANOVA test at a level of significance of *p* < 0.05. Note absence of significant differences between nuclei and treatments. *n* = 3 animals in each situation and nucleus.

For the study of laminin, we used the triple immunofluorescence sections (see section “Materials and Methods”) focusing on ChAT (in green) and laminin (in red). ChAT labeling was used to delimit the three motor nuclei of interest (see example for the oculomotor nucleus in **Figures 4A,D,G** for the control, axotomy and VEGF situations, respectively). The immunostaining against laminin yielded a very conspicuous signal outlining clearly the blood vessels throughout the histological sections in the different experimental conditions (**Figures 4B,E,H**). For the analysis, only those laminin-positive elements located within the limits of the nuclei outlined by the ChAT immunolabeling were considered (see merged images in **Figures 4C,F,I**). Using a grid (see section “Materials and Methods” for more details) we quantified the immunoreactivity against laminin in the abducens, trochlear and oculomotor nuclei for the control, axotomy and VEGF conditions. Axotomy and VEGF data were expressed as percentages relative to the control animals. For instance, in the case of the abducens nucleus, data were 100 ± 14.30%, 81.80 ± 18.16%, and 81.84 ± 8.19% for control, axotomized and VEGF-treated animals, respectively (*n* = 3 in each situation). As observed in **Figure 4J**, no significant differences were found in laminin immunostaining between either experimental conditions or motor nuclei (two-way ANOVA test).

**FIGURE 5:**
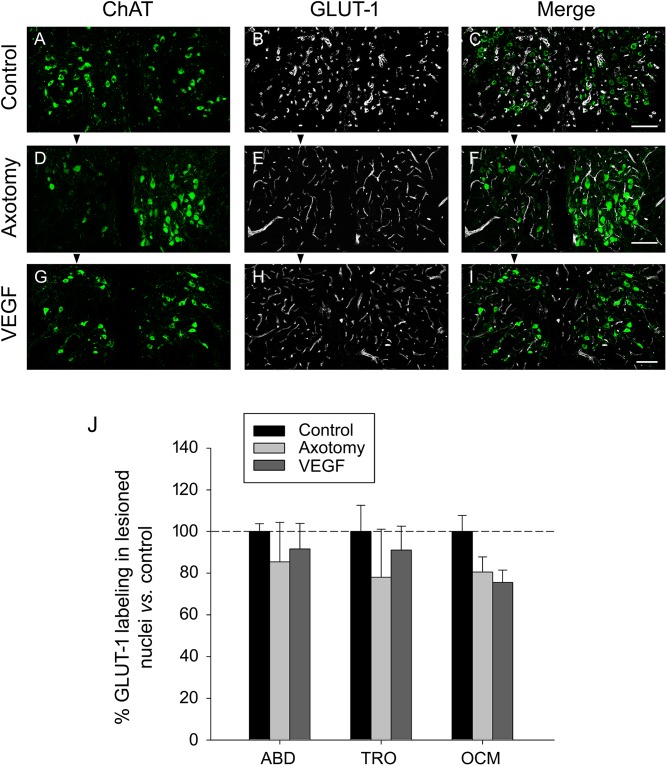
GLUT-1 immunostaining reveals absence of VEGF-induced angiogenesis. Confocal images of transverse sections through the oculomotor nucleus after double immunofluorescence against ChAT and GLUT-1. **(A–C)** Control intact animal. **(D–F)** Axotomized animal treated with vehicle. **(G–I)** Axotomized animal treated with VEGF. Arrowheads point to the affected (axotomized) side in **(D–I)**. Motoneurons were identified as ChAT-immunoreactive (**A,D,G**, in green). GLUT-1 was used as a marker of endothelial cells (**B,E,H**, in white). The merged images of ChAT and GLUT-1 are illustrated in **(C,F,I)**. Note a similar density of GLUT-1-labeled endothelial cells of blood vessels around motoneurons in the three experimental situations. Scale bars = 100 μm. **(J)** Bar chart illustrating the measurements of GLUT-1 immunoreactivity in the different experimental situations (control, axotomized and VEGF-treated animals) and for the three extraocular motor nuclei (ABD, abducens; TRO, trochlear; and OCM, oculomotor nuclei). Data represent mean ± SEM and are expressed relative to the control animals (100%, horizontal dashed line). Comparisons were carried out by means of the two-way ANOVA test at a level of significance of *p* < 0.05. Note absence of significant differences between nuclei and treatments. *n* = 3 animals in each situation and nucleus.

We also analyzed the selective marker of endothelial cells GLUT-1 in the triple immunofluorescence sections, according to the same procedure as described above for laminin. GLUT-1 (in white) was analyzed within the limits of the three extraoculomotor nuclei defined by the ChAT labeling (in green) and in the three experimental situations (control, axotomized, or VEGF-treated animals; *n* = 3 in each case). An example of GLUT-1 immunostaining for the oculomotor nuclei in the three situations is illustrated in **Figures 5A–I**. For quantification, data were normalized with respect to control animals (e.g., 100 ± 3.7%, 85.36 ± 18.99%, and 91.64 ± 12.24% for the abducens nucleus in control, axotomy and VEGF situations, respectively; *n* = 3 in each case). Comparison of the degree of GLUT-1 immunolabeling also revealed absence of significant differences between the different experimental conditions as well as between the different motor nuclei (two-way ANOVA test, **Figure 5J**). Altogether, the laminin and GLUT-1 data obtained in the present work pointed to absence of angiogenesis around the motoneurons following VEGF administration.

**FIGURE 6:**
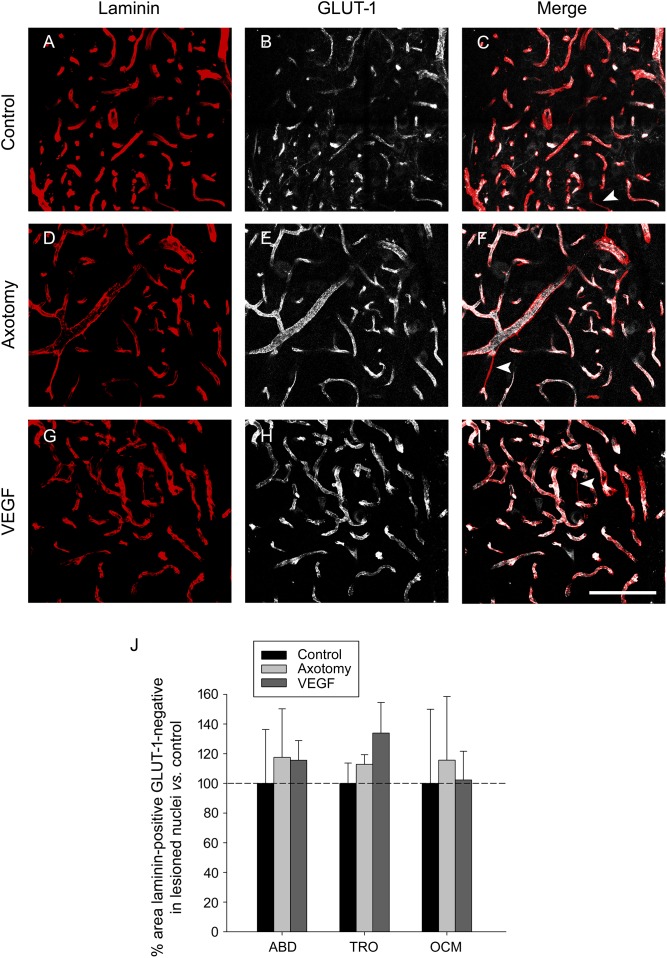
Labeling of blood vessels showing absence of angiogenic response to VEGF treatment. **(A,D,G)** Laminin immunostaining (in red) of the basement membrane of blood vessels in control **(A)**, axotomized **(D),** and VEGF-treated **(G)** animals. **(B,E,H)** Same as **(A,D,G)** but for GLUT-1 immunolabeling of endothelial cells (in white). The merged images of both markers are shown in **(C,F,I)** for control **(C)**, axotomy **(F)** and VEGF treatment **(I)**. Note similarity in blood vessel labeling in the three situations for the two markers. White arrowheads in **(C,F,I)** point to blood vessels which were laminin-immunopositive but GLUT-1-immunonegative, suggestive of newly formed vascular sprouts in the three situations. All images shown correspond to the oculomotor nucleus. Scale bar = 100 μm. **(J)** Bar chart illustrating the percentage of the area of blood vessels that was laminin-immunopositive but GLUT-1-immunonegative for the different experimental situations (control, axotomized, and VEGF-treated animals) and for the three extraocular motor nuclei (ABD, abducens; TRO, trochlear; and OCM, oculomotor nuclei). Data represent mean ± SEM and are expressed relative to the control animals (100%, horizontal dashed line). Comparisons were carried out by means of the two-way ANOVA test at a level of significance of *p* < 0.05. Note absence of significant differences between nuclei and treatments. *n* = 3 animals in each situation and nucleus.

Since GLUT-1 labels mature endothelial cells (Krum et al., 2002), we performed another analysis in the triple immunostained sections consisting in the measurement of the area of those laminin-outlined blood vessels which lacked any associated GLUT-1 immunostaining (**Figure 6**), assuming that these structures could possibly represent immature newly-formed vessels (arrowheads in **Figures 6C,F,I**). We carried out the analysis in the abducens, trochlear and oculomotor nuclei of control, axotomized and VEGF-treated animals (*n* = 3 animals in each situation). The statistical analysis of laminin-positive, GLUT-1 negative areas, normalized to control, revealed that there were no significant differences in the values obtained between either the different experimental situations or the three motor nuclei (two-way ANOVA test). These findings reinforce those obtained after the analysis of laminin and GLUT-1 carried out independently (see above). Therefore, according to the findings obtained with all vascular markers used in the present work (albumin, laminin and GLUT-1), it might be propose that VEGF, at this dose, did not induce in the extraoculomotor nuclei any increase in the vascular network or in permeability that could support the neuroprotective effect exerted by this factor on the cholinergic phenotype of severed motoneurons. Accordingly, our data point to a direct action of VEGF on neurons.

## Discussion

The present work has demonstrated that the exogenous administration of VEGF is able to maintain injured motoneurons with a normal level of ChAT, the biosynthetic enzyme of their neurotransmitter acetylcholine, thereby preventing the downregulation in ChAT that ensues motoneuronal lesion. The fact that ChAT can be maintained in axotomized VEGF-treated motoneurons likely implies a normal level of the motoneuronal neurotransmitter acetylcholine. Consequently, this finding is of great interest since it would imply that VEGF can sustain lesioned motoneurons in an operational state, at least regarding the synthesis of neurotransmitter as a first step necessary for neuromuscular transmission. Therefore, our data strongly support the potentiality of VEGF as a therapeutic tool for the treatment of motoneuronal diseases, reinforcing previous findings showing that this factor can prevent motoneuronal death in animal models of ALS (Azzouz et al., 2004; Storkebaum et al., 2005; Wang et al., 2007; Sathasivam, 2008). The present data add the possibility that this factor might also maintain surviving motoneurons in a neurotransmissive mode. Moreover, our evaluation of the vasculature and capillary permeability in the extraocular motor nuclei indicated that the neuronal effects mediated by VEGF are likely the consequence of a direct action of this factor on the motoneurons, rather than an indirect effect mediated by the angiogenic activity of VEGF.

### ChAT Expression Is Downregulated in Motoneurons After Lesion or Disease

A general phenomenon described in motoneurons following axotomy is ChAT downregulation. Previous studies in hypoglossal, facial and spinal motoneurons have shown that, after nerve lesion, damaged motoneurons lose their immunoreactivity for ChAT and express low levels of the transcript (Yan et al., 1994; Friedman et al., 1995; Tuszynski et al., 1996; Matsuura et al., 1997; Wang et al., 1997). We have also reported previously the loss of the cholinergic phenotype in extraocular motoneurons after axotomy, both in adult and neonatal rats, with the difference that motoneurons finally die after axotomy in the case of postnatal animals but they survive in adulthood (Morcuende et al., 2005, 2013). ChAT downregulation reaches its maximum by 7 days postlesion, for the motoneurons of the three extraocular motor nuclei (Morcuende et al., 2005). For this reason we performed the present work at the time point when lesion-induced effects on ChAT expression were maximum, in order to determine whether VEGF administration could be effective in the most drastic conditions.

Injured motoneurons not only exhibit a downregulation in ChAT but also many other metabolic, biochemical, and morphophysiological changes (Titmus and Faber, 1990; Navarro et al., 2007; Allodi et al., 2012). For instance, whereas molecules related with neurotransmission decrease, cytoskeleton and structural proteins increase. These changes has been interpreted as a switch in motoneuron function from a neurotransmissive role to a regenerative mode after their lesion (Moran and Graeber, 2004; Morcuende et al., 2005, 2013; Navarro et al., 2007).

It is important to point out that ChAT immunoreactivity is also markedly reduced in large spinal neurons in ALS cases (Oda et al., 1995). In addition, the activity and mRNA of this enzyme are also decreased in patients with ALS (Nagata et al., 1982; Virgo et al., 1992). Indeed, preserved neurons in the anterior horn of ALS spinal cord from early non-advanced stages of the disease show a lower ChAT activity than control neurons (Kato, 1989), whereas there are no changes in other proteins (Clark et al., 1990). All these findings have raised the suggestion that low expression of ChAT is a specific and early change in the pathogenesis of ALS (Oda, 1999).

### VEGF Sustains the Cholinergic Phenotype in Damaged Motoneurons

The present results have demonstrated that VEGF administered exogenously immediately after lesion is able to prevent the loss of the cholinergic phenotype in axotomized motoneurons by inhibiting the drop in the levels of the enzyme ChAT, and hence, probably, allowing motoneurons to synthetize their neurotransmitter acetylcholine. Moreover, we found similar results in the three extraocular motor nuclei (i.e., abducens, trochlear, and oculomotor).

The finding that VEGF maintains the level of ChAT expression in damaged motoneurons likely reflects a normal synthesis of acetylcholine in the motoneurons, which would be of great functional relevance for the proper neuromuscular communication. As stated above, several experiments have demonstrated that the administration of VEGF may alleviate motoneuronal degeneration in ALS animal models (Azzouz et al., 2004; Storkebaum et al., 2005; Tovar-y-Romo et al., 2007; Dodge et al., 2010; Tovar-y-Romo and Tapia, 2010) and that ChAT is also downregulated in ALS patients (Nagata et al., 1982; Kato, 1989; Clark et al., 1990; Virgo et al., 1992; Oda et al., 1995; Oda, 1999). According to those data and our present findings, the possibility exists that VEGF applied to diseased motoneurons not only rescues them from cell death but also maintains their neurotransmissive phenotype.

Previous studies have shown that other neurotrophic factors are also able to prevent the loss of the cholinergic phenotype in axotomized motoneurons. Thus, we have previously applied brain-derived neurotrophic factor, nerve growth factor, glial cell-line-derived neurotrophic factor or neurotrophin-3 to neonatal rats enucleated at postnatal day 0, using the same procedures as in the present work. The results obtained showed that all these factors rescue postnatal axotomized motoneurons from cell death and that all, except NT-3, also prevent the reduction in ChAT subsequent to axotomy (Morcuende et al., 2013). In addition to extraocular motoneurons, other types of motoneurons are also protected from the lesion-induced loss of ChAT after the administration of different neurotrophic factors (Yan et al., 1994, 1995; Friedman et al., 1995; Tuszynski et al., 1996; Wang et al., 1997; Fernandes et al., 1998; Sakamoto et al., 2003; Zhao et al., 2004). However, although these other molecules have also shown the capability to prevent the downregulation in ChAT, or other aspects of motoneuron physiology, their lack does not cause adult-onset ALS-like motoneuron degeneration and paralysis in mice, as happens for VEGF (Oosthuyse et al., 2001; Lambrechts and Carmeliet, 2006; Gould and Oppenheim, 2011; Tovar-y-Romo et al., 2014). In addition, VEGF is probably the most potent among all neurotrophic factors tested in experimental ALS models (Gould and Oppenheim, 2011; Tovar-y-Romo et al., 2014) and the only one showing an evident relationship between low levels of the factor (and/or its main receptor VEGFR-2) and motoneuron degeneration, as shown in both animal models and human cases of ALS (Oosthuyse et al., 2001; Brockington et al., 2006; Sathasivam, 2008).

Recently, we have shown that the administration of VEGF to axotomized abducens motoneurons in cats prevents and recovers the discharge characteristics and synaptic complement that are typically affected by lesion (Calvo et al., 2018). Together with the present data, indicating the maintenance of the neurotransmitter biosynthetic enzyme in VEGF-treated axotomized extraocular motoneurons, it can be suggested that VEGF likely acts as an important, crucial neurotrophic factor for motoneurons of the oculomotor system. This assumption can be extended to other motoneuronal types, given that the deficit in VEGF present in the mutant mice VEGF^δ/δ^ leads to motoneuron degeneration in the spinal cord (Oosthuyse et al., 2001). Altogether, it seems that VEGF could be also a powerful neurotrophic factor for other non-extraocular motoneurons.

### Evidences of Direct Versus Angiogenic Effects of VEGF on Motoneurons

Two mechanisms have been proposed to explain the neuroprotective action of VEGF on neurons exposed to different types of insult or disease (Oosthuyse et al., 2001; Carmeliet and Storkebaum, 2002; Storkebaum et al., 2004; Lange et al., 2016). First, due to its well-known activity on blood vessels, the promotion of vascular proliferation and permeability, a likely mechanism of action for exogenously applied VEGF, could lead to an increased vascular perfusion within the neural tissue, providing an extra supply of oxygen and nutrients to neurons which would contribute to their recovery. The second possibility is that VEGF acts directly on neurons activating signaling pathways that finally lead to the neuroprotective effects described for this factor. Both mechanisms could indeed coexist (Oosthuyse et al., 2001; Carmeliet and Storkebaum, 2002; Storkebaum et al., 2004; Lange et al., 2016).

There is considerable evidence in favor of a direct, receptor-mediated, protective action of VEGF on neurons (Jin et al., 2001; Matsuzaki et al., 2001; Azzouz et al., 2004; Tovar-y-Romo et al., 2007; Nicoletti et al., 2008; Tovar-y-Romo and Tapia, 2010; Lladó et al., 2013) and some works describe absence of vascular effects after VEGF exogenous administration (Azzouz et al., 2004; Tovar-y-Romo et al., 2007; Nicoletti et al., 2008). On the other hand, some studies have revealed that VEGF can induce angiogenesis (Krum et al., 2002) or increased permeability (Croll et al., 2004) and that its deficit can lead to decreased neural vascular perfusion (Oosthuyse et al., 2001). Therefore, since there is not at present a clear consensus on the possibility of vascular effects following VEGF administration, we were interested in evaluating a possible angiogenic response in our model that could mediate the VEGF-induced ChAT recovery.

In the present work, we have used laminin as a marker of the basement membrane of blood vessels and GLUT-1 as a specific marker of endothelial cells to analyze whether VEGF administration could promote abnormal growing of vascular structures that could supply more oxygen and nutrients to the affected motoneurons. Moreover, since VEGF also increases vascular permeability, we have also used albumin to check the possibility of extravascular leakage. These markers have also been used previously by other authors (Oosthuyse et al., 2001; Krum et al., 2002; Azzouz et al., 2004). Our data have shown that the intensity of immunostaining against laminin and GLUT-1 in the affected side of the abducens, trochlear, and oculomotor nuclei was similar in the axotomized animals, treated either with vehicle or VEGF, as compared to the control group (intact animals). The same result was obtained with the analysis of albumin immunolabeling. However, regarding albumin, it should be pointed out that, due to its high molecular weight, it might be not suitable for detecting small fluid leakage. Nevertheless, and in agreement with previous works (Oosthuyse et al., 2001; Krum et al., 2002; Azzouz et al., 2004), our findings showed that the administered VEGF was not accompanied by either angiogenesis or a significantly increased vascular permeability around treated motoneurons. Therefore, it could be assumed that the action of this factor on ChAT expression was likely due to a direct effect on the motoneurons, instead of an indirect effect due to increased blood perfusion.

Since GLUT-1 labels mature endothelial cells (Krum et al., 2002), we also quantified those regions of blood vessels, identified as laminin-immunoreactive, which lacked GLUT-1 labeling, as likely indicative of newly-formed vessels. Our analysis also revealed absence of differences between the three groups (control, axotomized, and VEGF-treated animals), which seems to indicate again that VEGF administration did not activate angiogenesis in the tissue surrounding extraocular motoneurons, in agreement with previous data using the same procedure (Krum et al., 2002).

It is important to stand out that the differences found in the literature regarding vascular *versus* neuronal effects following VEGF exogenous application could be due to two major factors. First of all, the dose employed might contribute to vascular effects when VEGF is applied at high doses. Thus, a dose-dependent study in ischemic rat brain has shown that low (2 μg/7 days) and intermediate (8 μg/7 days) doses of VEGF are not followed by angiogenesis, whereas a high dose (60 μg/7 days) does induce a powerful angiogenic response (Manoonkitiwongsa et al., 2004). Since we used a Gelfoam implant containing 1 μg of VEGF that was left *in situ* by 7 days, our dose was at the lower range of the dosages used by Manoonkitiwongsa et al. (2004). Moreover, those authors applied VEGF through the internal carotid, which represents a more specific and direct route of delivery as compared with our Gelfoam pellet implant applied peripherally in the enucleated orbit; therefore our “effective” VEGF dose reaching the brainstem was likely lower. The second factor that could influence an angiogenic VEGF-mediated activity is the pathway of administration. Thus, Krum et al. (2002) have described a higher vascular proliferation in adult rat brain using intracerebral osmotic minipump than after the less traumatic subdural gelatine sponge placement. In the present work, we applied VEGF from the periphery (intraorbitally) without interfering with the integrity of the central nervous system and, therefore, reducing the risk of promoting angiogenic activity in the brain. Indeed, we have previously shown that extraocular motoneurons express the receptor VEGFR-2 in rats (Silva-Hucha et al., 2017; VEGFR-1 not tested), and in the cat the two receptors VEGFR-1 and VEGFR-2 are present (Calvo et al., 2018). Moreover, VEGF has been previously shown to be retrogradely transported along axons (Storkebaum et al., 2005). Therefore, given that in our study VEGF was administered through a gelatine sponge implanted into the orbit after enucleation, this factor likely reached motoneurons through retrograde transport from the proximal stumps of sectioned extraoculomotor axons and not through the vasculature surrounding motoneurons.

Summing up, the dose and experimental procedure that we used for VEGF delivery were in the direction of minimizing any vascular action of VEGF in the brain. Moreover, the quantification of vascular vessels, endothelial cells and albumin indicated absence of significant changes in the vasculature and permeability after VEGF treatment. Altogether, the present data point to a direct action of VEGF on extraocular motoneurons which prevented the lesion-induced decrease in ChAT. Consequently, VEGF sustained these damaged motoneurons in a neurotransmissive mode, which is a necessary prerequisite for neuromuscular function, opening promising perspectives for the use of VEGF as a therapeutical agent in motoneuron diseases.

## Author Contributions

RdC designed the experiments. LA, SS-H, SM, and AP performed the experiments. LA, SS-H, and SM analyzed the data. RdC and AP wrote the manuscript. All authors contributed to manuscript revision, read and approved the submitted version.

## Conflict of Interest Statement

The authors declare that the research was conducted in the absence of any commercial or financial relationships that could be construed as a potential conflict of interest.
